# A Systematic Review and Meta-Analysis Comparing FAST and BEFAST in Acute Stroke Patients

**DOI:** 10.3389/fneur.2021.765069

**Published:** 2022-01-28

**Authors:** Xinjie Chen, Xiaoxiao Zhao, Fan Xu, Mingjin Guo, Yifan Yang, Lianmei Zhong, Xiechuan Weng, Xiaolei Liu

**Affiliations:** ^1^Department of Neurology, The First Affiliated Hospital of Dali University, Dali, China; ^2^Department of Neurology, The First Affiliated Hospital of Kunming Medical University, Kunming, China; ^3^Yunnan Provincial Clinical Research Center for Neurological Diseases, Kunming, China; ^4^Department of Public Health, Chengdu Medical College, Chengdu, China; ^5^Department of Vascular Surgery, The Affiliated Hospital of Qingdao University, Qingdao, China; ^6^Department of Pain Medicine, Peking University Peoples Hospital, Beijing, China; ^7^Department of Neuroscience, Beijing Institute of Basic Medical Sciences, Beijing, China

**Keywords:** stroke, acute stroke, FAST, BEFAST, meta-analysis, systematic review

## Abstract

**Objective:**

To evaluate and compare the predictive value of Face, Arm, Speech Test (FAST) and Balance, Eyes, Face, Arm, Speech, Time (BEFAST) scale in the acute ischemic stroke (AIS).

**Methods:**

We searched Medline and Ovid databases for relevant literature in the English language. There were no limitations on the date. The sensitivity, specificity, likelihood ratio, and diagnostic odds ratio were pooled for meta-analysis. The symmetric receiver operator characteristic curve and Fagan's Nomogram were drawn, and meta-regression and subgroup analysis were used to explore the source of heterogeneity.

**Results:**

A total of 9 studies, including 6,151 participants, were analyzed. The combined sensitivity of FAST was 0.77 [95% *CI* (0.64–0.86)], specificity was 0.60 [95% *CI* (0.38–0.78)], the area under the ROC curve was 0.76, and the diagnostic ratio was 1.57, while the sensitivity of BEFAST was 0.68 [95% *CI* (0.23–0.93)], specificity was 0.85 [95% *CI* (0.72–0.92)], the area under the ROC curve was 0.86, and the diagnostic odds ratio was 2.44. No publication bias was detected in Deeks' funnel plot. For FAST, meta-regression analysis showed that the prospective design, satisfactory description of the index test, and a broad spectrum of disease contributed to the heterogeneity in sensitivity, while no sources contributed to the heterogeneity in sensitivity. When the pretest probability was set as 20%, the posterior probability in Fagan's Nomogram was 32%; however, when the pretest probability was set as 20% in BEFAST, the posterior probability in Fagan's Nomogram was 52%.

**Conclusions:**

Our findings indicated that FAST and BEFAST might be useful in the diagnosis of acute ischemic stroke. The diagnostic value of BEFAST in acute ischemic stroke was higher than in FAST; thus, it might have an important role in the fast recognition of acute ischemic stroke.

## Introduction

Stroke is one of the most common acute and severe diseases presented to an emergency department (ED). Stroke is a major global burden, with 10.3 million new strokes and 113 million disability-adjusted life years (DALYs) per year worldwide (1). It can be divided into transient ischemic attack (TIA), ischemic stroke (IS), hemorrhagic stroke (HS), and subarachnoid hemorrhage. Acute ischemic stroke (AIS) can have serious lifelong consequences. In contrast to HS, significantly improved survival in IS patients has been reported since the early 2000s (2). Therefore, early recognition of stroke is of utmost importance. Rapid recognition of stroke warning signs is a crucial factor in the acute treatment of stroke. Prehospital stroke scales are helpful to guide the prehospital selection of people suspected of having a stroke (3). The screening tools can develop to help the public recognize stroke early. Earlier and improved stroke detection by ED and ambulance may reduce treatment delays (4).

Insufficient knowledge on stroke among the general public may lead to serious consequences. Several screening tools, including the Cincinnati Prehospital Stroke Scale (CPSS), Face, Arm, Speech Test (FAST), Los Angeles Prehospital Stroke, Screen (LAPSS), Melbourne Ambulance Stroke Screen (MASS), Medic Prehospital Assessment for Code Stroke (Med PACS) and Recognition of Stroke in the Emergency Room score (ROSIER), which are characterized by simple, structured, and easy-to-use stroke recognition scores, have been developed to help the public identify if a person is having an acute stroke so as to facilitate rapid access to medical care. Among these scales, the FAST provides the highest sensitivity with 85%. However, the available stroke recognition scores have a huge variety of length and complexity, which complicates choosing the optimal score in the emergency setting (5). Furthermore, it is truly difficult to compare the reported diagnostic accuracies of recognition scores. The FAST fails to detect 40% of those with posterior circulation events, especially those with ataxia and visual disturbances (6). A previous study showed that “FAST” failed in 14% of AIS patients (7).

BEFAST (Balance, Eyes, Face, Arm, Speech, Time), which was previously studied to determine whether adding gait or visual abnormalities to the FAST scale would improve stroke detection rates, revealed statistically lower Sensitivity for the detection of AIS in the inpatient population compared with the ED (8). However, a prospective study in 2018 has shown that BEFAST assessment does not improve stroke detection in the prehospital setting (4).

It is necessary to improve the accuracy of scales. This systematic review and meta-analysis aimed to explore the diagnostic value of the FAST and BEFAST for AIS patients; a quantitative reference for clinical practice was provided.

## Methods

### Search Strategy

Two reviewers (CXJ and ZXX) independently searched the PubMed, Embase, and Cochrane libraries for all the relevant publications published thus far. We chose the keywords “stroke,” “ischemic stroke,” and “hemorrhagic stroke” as text words and MeSH terms to identify related studies, language, region, or publication type. The search was limited to published clinical studies. Search terms are listed as follows:

(FAST)[Title/Abstract]
*(BEFAT)[Title/Abstract]*
*1 OR 2*
*(“stroke” or “ischemic stroke” or “hemorrhagic stroke”) [Title/Abstract]*
*3 AND 4*
*From 2011 to 2021*

### Inclusion and Exclusion Criteria

Inclusion criteria were: (1) all types of strokes; (2) included FAST or/and BEFAST; (3) clinical study; (4) published within past 10 years; and (5) published in the English language.

Exclusion criteria were: (1) no described outcomes; (2) no control groups; (3) impossible to find original paper; and (4) the sensitivity, specificity (Sp), positive predictive value, and negative predictive value cannot be extracted.

### Data Extraction

Two authors (CXJ and ZXX) independently extracted the demographic data and treatment information, and if a disagreement occurred, a third author (XF) was involved. Baseline information extracted from 9 studies contained the first author name, year of publication, title, design type, study subjects (number, age, male/female ratio), disease degree, and length of the disease. Besides, the primary outcomes included True positives (Tp), False positives (Fp), False negatives (Fn), true negatives (Tn) with FAST and BEFAST.

### Quality Assessment

The Agency for Healthcare Research and Quality (AHRQ) was used to rate the methodological quality of cross-sectional studies. An item was scored with “0” if it was answered “NO” or “UNCLEAR”; if it was answered “YES,” then it was scored “1.” Article quality was assessed as follows: low quality = 0–3; moderate-quality = 4–7; and high quality = 8–11. The quality of studies was assessed by using the Newcastle Ottawa scale (NOS), which generated a maximum of nine stars for each study, including four stars for the selection of participants, two stars for the comparability of participants, and three stars for the assessment of outcomes. Quality was assigned according to the final scores, where 7–9 stars indicated high quality, 4–6 stars for middle quality, and 0–3 stars for low quality (9).

### Statistical Analysis

Stata 15.0 software (Stata Corp 4905 Lakeway Drive, College Station, TX, USA) was used to perform a meta-analysis. The bivariate model was used to calculate the combined Sensitivity (Se), Specificity (Sp), the positive likelihood ratio (PLR), the negative likelihood ratio (NLR), and diagnostic odds ratio (DOR), and to draw the symmetric receiver operator characteristic curve (SROC) so as to estimate the total diagnostic accuracy. Pre-test probabilities may be estimated from routine data, practice data, or clinical judgment. Post-test probabilities are used to determine whether the probability of diagnosis has raised or fallen, compared with pre-test probabilities. The heterogeneity was assessed by Cochrane's *Q* statistics (chi-square), or inverse variance (*I*^2^). *I*^2^ <50% and *p* > 0.1 indicated that these studies could be considered as homogeneous by using a fixed-effect model; otherwise, *I*^2^ ≥ 50%, *p* <0.10, the random effect model, was used for meta-analysis. If heterogeneity among studies was recorded, the potential source of heterogeneity was investigated *via* meta-regression. A *p* value <0.05 was considered statistically significant.

## Results

### Flowchart and Study Quality

**Figure 1:**
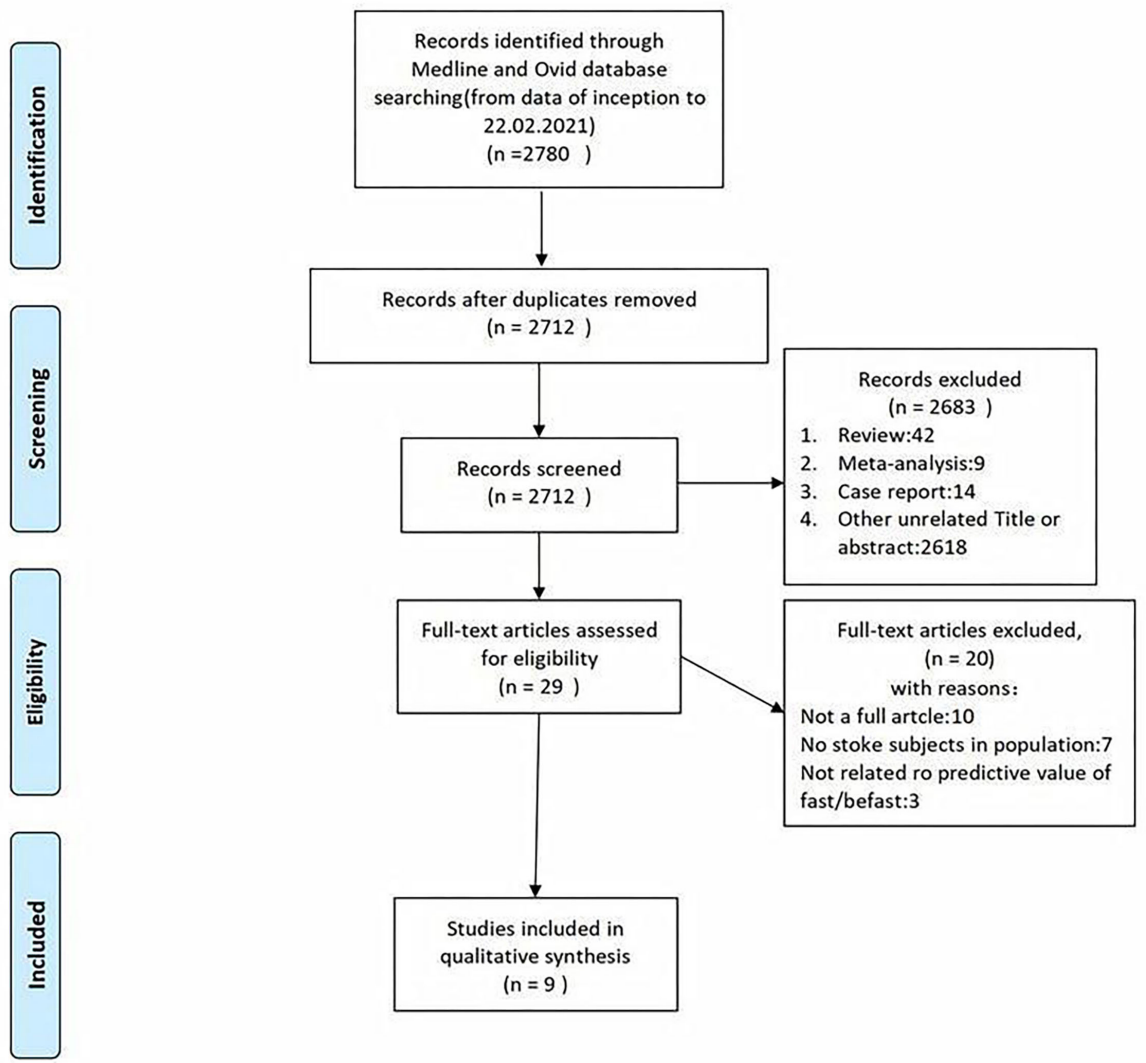
The literature with Face Arm Speech Test (FAST) and Balance, Eyes, Face, Arm, Speech, Time (BEFAST) screening process of the meta-analysis.

A total of 7,690 papers with FAST and BEFAST (including documents, reviews, animal experiments, case reports, and repeated studies) were retrieved from each database. After 1,825 duplicate records were removed, the full text of the remaining 5,865 studies was read. Among those studies, 201 were excluded because the articles were reviews, meta-analyses, or case reports, while 5,642 studies did not have related titles and abstracts. The full text of the remaining 21 studies was read, and 12 studies were removed due to incomplete data. The remaining 9 papers were extracted from the corresponding data according to the data extraction requirements. Seven studies used the FAST; one study used the BEFAST and one study used the FAST and BEFAST. The literature screening process is shown in Figure 1. The basic characteristics and inclusive and exclusive criteria of each included study are shown in Tables 1, 2.

**Table 1:** Basic characteristics of enrolled studies.

**Author**	**Study**	**Year**	**Region**	**number**	**Age (mean ±sd)**	**Sex (male%)**	**Scale used**	**Outcome**
D. Václavík (10)	Prospective cohort	2018	Czech	435	74 ± 12	215 (51.0)	FAST-plus	The sensitivity, specificity, positive predictive value, negative predictive value of the FAST plus test in detecting LVO stroke.
S. Aroor (7)	Cross-sectional	2017	American	736	NA	NA	FAST/BEFAST	Missed diagnosis rate of the FAST or BEFAST in detecting stroke.
D. Pickham (4)	Prospective cohort	2018	American	359	NA	Stroke: 55 (34.6); Non-stroke: 46 (23.0)	FAST	The sensitivity, specificity of the diagnosis of stroke after using FAST or BEFAST.
H. Mao (11)	Prospective cohort	2016	China	416	Stroke (*n* = 358): 69.2 ± 13.8; non-stroke (*n* = 58): 70.6 ± 11.4	Stroke: 210 (58.7): non-stroke:37 (63.8)	FAST	The sensitivity, specificity of the diagnosis of stroke after using FAST.
RT. Fothergill (12)	Prospective cohort	2013	UK	295	65	156 (53.0)	FAST	The sensitivity, specificity, positive predictive value, negative predictive value of the FAST plus test in detecting stroke.
A. Berglund (13)	Prospective cohort	2014	Sweden	900	71	NA	FAST (EMCC) FAST (Ambulance)	The positive predictive values (PPV) for a stroke/TIA diagnosis at discharge after using FAST.
JC. Purrucker (5)	Prospective cohort	2015	Germany	689	Total (*n* = 689): 61.7 ± 20.9; Stroke (*n* = 00): 75.6 ± 13.4; non-stroke (*n* = 489): 56.0 ± 20.8	Total: 357 (51.8); Stroke: 80 (40.0); non-stroke: 277 (56.6)	FAST	The sensitivity, specificity, positive predictive value, negative predictive value of the FAST plus test in detecting stroke.
WN Whiteley (14)	Prospective cohort	2011	UK	356	NA	173 (48.6)	FAST	The sensitivity, specificity of the diagnosis of stroke or TIA after using FAST.
F. El Ammar (8)	Cross-sectional	2020	American	1965	Total: 63 ± 16.1; In-hospital stroke: 61.6 ± 17.3; Prehospital/ED stroke: 63.3 ± 15.6	Total: 844 (43); In-hospital stroke: 232 (47.4); Prehospital/ED stroke: 612 (41.5)	BEFAST (All patients); BEFAST (prehospital/ED)	The sensitivity, specificity of the diagnosis of stroke after using BEFAST.

*NA, not available from original study paper or supplementary or registration information; ED, emergency department; LVO, large vessel occlusion. EMCC, Emergency Medical Communication Center*.

**Table 2:** Inclusion and exclusion criteria.

**Author**	**Inclusion criteria**	**Exclusion criteria**	**Scale**
D. Václavík (10)	(a) Suspected acute stroke patient admitted to one of the three-stroke centers; (b) FAST PLUS test evaluation by paramedics; and (c) CT and CTA evaluations.	The exclusion criterion was suspected stroke with more than 12 h from symptom onset.	FAST-plus
S. Aroor (7)	Patients with a discharge diagnosis of acute ischemic stroke (International Classification of Diseases, Ninth Revision, Clinical Modification codes) were reviewed.	Those misclassified, having missing NIHSS data, or were comatose or intubated were excluded. Presenting symptoms, demographics, and examination findings based on the NIHSS were abstracted.	FAST
D. Pickham (4)	NA	NA	FAST
H. Mao (11)	Suspected stroke patients ≥18 years old presenting to the ED with symptoms or signs within 7 days were recruited.	Patients were excluded if they were <18 years old, had a traumatic brain injury, subarachnoid hemorrhage, or unknown diagnosis.	FAST
RT. Fothergill (12)	Aged >18 years if they presented with symptoms of stroke, were assessed by participating ambulance clinicians using the ROSIER, and conveyed to the Royal London Hospital.	We did not include those who were <18 years, not assessed using the ROSIER, or transferred to another hospital.	FAST
A. Berglund (13)	The study population consisted of all calls to the EMCC concerning patients presenting at least one FAST symptom or a history/finding, making the EMCC or ambulance personnel suspect a stroke within 6 h.	NA	FAST (EMCC) FAST (Ambulance)
JC. Purrucker (5)	we selected consecutive cases allocated to the database category “suspected central nervous system disorder,” that is, patients with potential stroke and stroke-mimics.	Excluding repeated and primary neurotrauma admissions and cases with missing discharge diagnosis.	FAST
WN Whiteley (14)	(a) whose symptoms began <24 h before admission, (b) who were still symptomatic at the time of assessment and (c) in whom a general practitioner, a paramedic or a member of the emergency-department staff had made a diagnosis of “suspected stroke.”	NA	FAST
F. El Ammar (8)	(a) age 18 year or older; (b) PH stroke alert activation by emergency medical personnel enroute to the ED, stroke activation by ED staff members, or in-hospital stroke alert activation.	(a) age 17 years or younger; (b) cancellation of stroke alert activation by the primary team prior to arrival of the stroke response team; (c) conversion of stroke alert to cardiac arrest code at time of arrival of stroke response team, (d)missing data at time of chart review.	BEFAST (All patients); BEFAST (prehospital/ED)
S. Aroor (7)	Patients with a discharge diagnosis of acute ischemic stroke (International Classification of Diseases, Ninth Revision, Clinical Modification codes) were reviewed.	Those misclassified, having missing NIHSS data, or were comatose or intubated were excluded. Presenting symptoms, demographics, and examination findings based on the NIHSS were abstracted.	BEFAST
D. Pickham (4)	Patients with sudden onset of neurological symptoms <6 h from EMS arrival were assessed with BEFAST in the field.	NA	BEFAST

*NA, not available from original study paper or supplementary or registration information; FAST, Face Arm Speech Test; BEFAST, Balance, Eyes, Face, Arm, Speech, Time; NIHSS, National Institutes of Health Stroke Scale; ED, emergency department; ROSIER, Recognition of Stroke in the Emergency Room score; EMCC, Emergency Medical Communication Center*.

### FAST Against AIS

**Figure 2:**
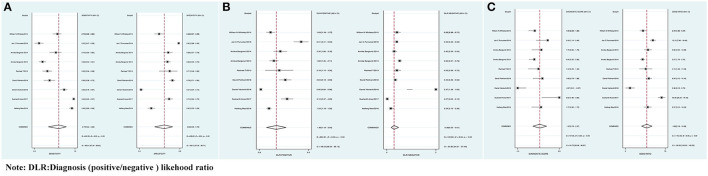
**(A)** Forest plot of sensitivity (Se) and specificity (Sp) of FAST in the diagnosis of acute ischemic stroke (AIS). **(B)** Forest plot of diagnosis (positive/negative) likehood ratio (DLR) positive and negative of AIS. **(C)** Forest map of the diagnostic odds ratio (DOR) of FAST in the diagnosis of AIS.

The combined Se of FAST in AIS was 0.77 [95% *CI* (0.64, 0.86)], Sp was 0.60 [95% *CI* (0.38, 0.78)], PLR was 1.90 [95% *CI* (1.18, 3.04)], NLR was 0.39 [95% *CI* (0.25, 0.61)], area under ROC curve was 0.76, and DOR was 4.82, which indicated the FAST had a medium value in the screen of AIS. As all heterogeneity w*as I*^2^ > *50%*, the random model was used. The details of the combined Se and Sp forest plot are shown in Figure 2A, the combined likelihood ratio forest plot in Figure 2B, and the combined diagnosis ratio forest plot in Figure 2C.

### Publication Bias

**Figure 3:**
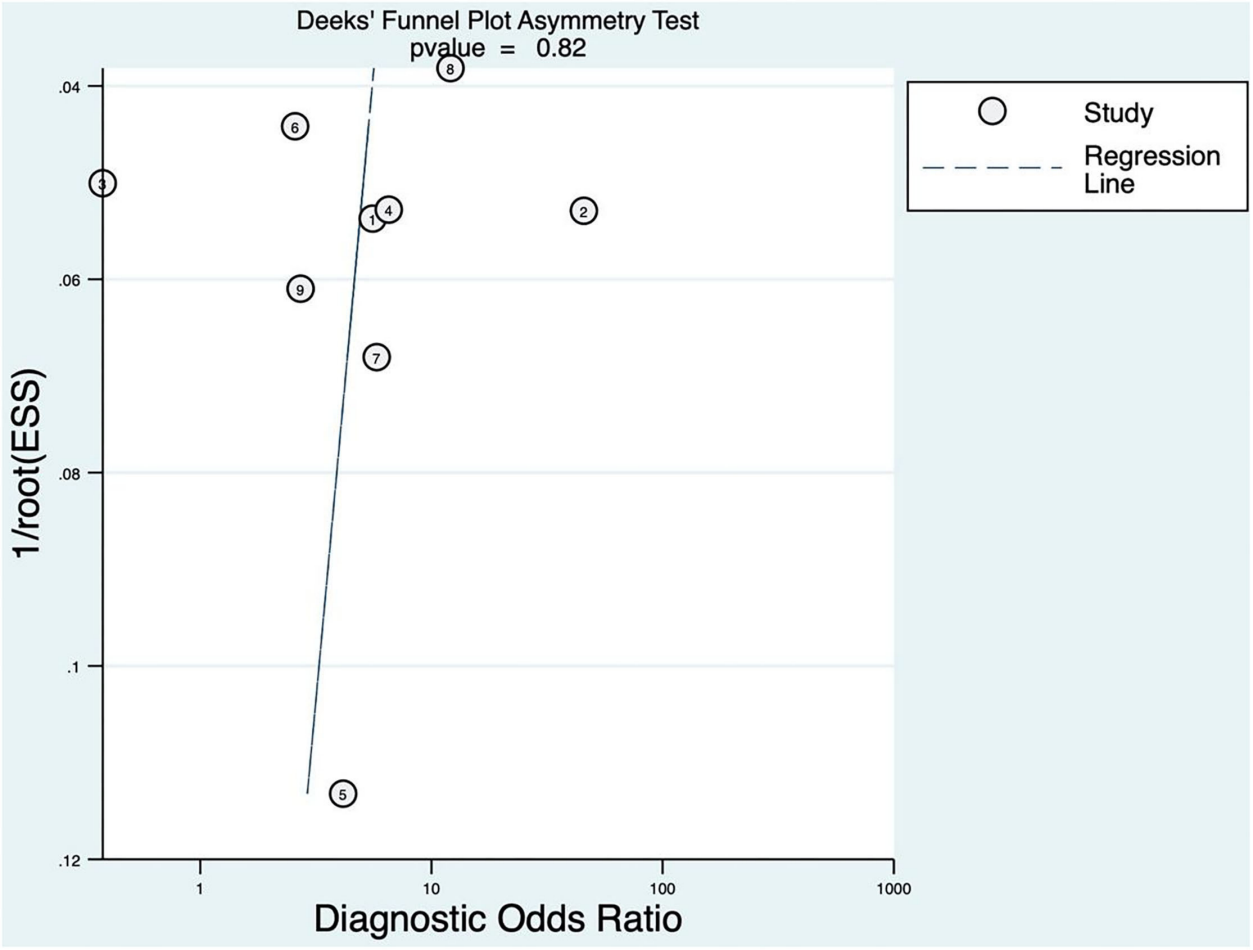
Deeks' funnel plot asymmetry test for FAST.

The linear regression was used to test funnel asymmetry so as to evaluate publication bias. The results showed no asymmetry, while the linear regression test *p* was 0.82, which indicated no publication bias, as shown in Figure 3.

### Threshold Effect

**Figure 4:**
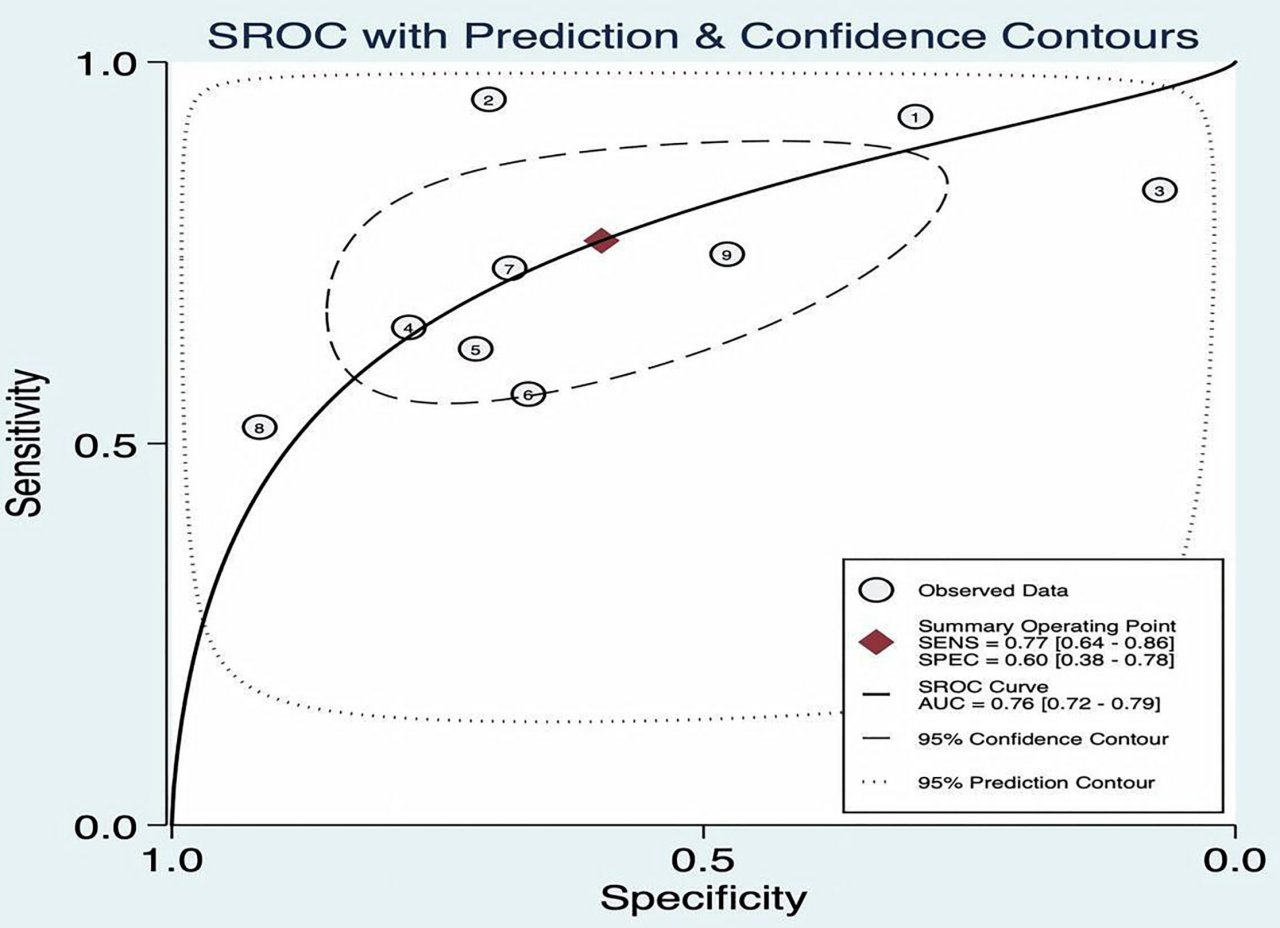
Summary receiver operating characteristic (ROC) of FAST.

The SROC curve plane test was used to threshold effect. However, there was no typical “shoulder arm” found, indicating no threshold effect. Moreover, Cochran's Q value was 59.49, and the *p* was <0.05, which indicated that the heterogeneity was caused by the non-threshold effect. A moderate diagnostic value could be concluded by the value of the area under the SROC curve (AUC), which was 0.76 [95% *CI* (0.72–0.79)], as shown in Figure 4.

### Meta-Regression and Subgroup Analysis

**Figure 5:**
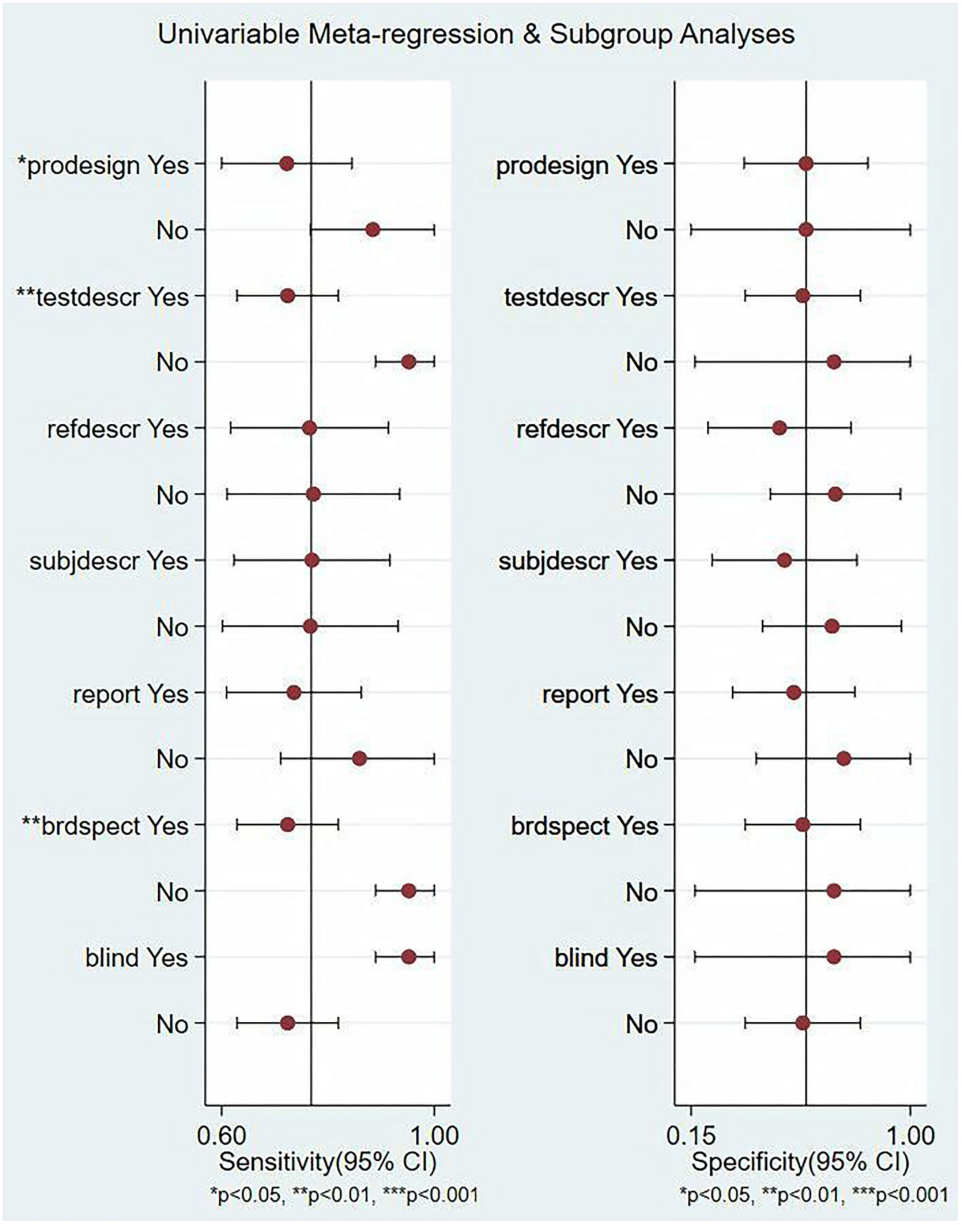
Single-factor meta-regression and subgroup analysis. Prospective design: prodesign, testdescr: satisfactory description of the index test, subjdescr: adequate description of study subjects, refdescr: satisfactory description of ref test, and brdspect: broad spectrum of disease^1^.

In this study, we evaluated the factors that may affect the heterogeneity, such as non-threshold effect, prospective design (prodesign), satisfactory description of index test (testdescr), an adequate description of study subjects (subjdescr), satisfactory description of ref test (refdescr), report, a broad spectrum of disease (brdspect), and whether the test results were evaluated by a blind method. The meta-regression analysis of the above factors revealed that although the sources of heterogeneity of Se were statistically related to the prodesign, testdescr, and brdspect, the sources of heterogeneity of Sp were not related to these factors, as shown in Figure 5.

### Pre-test Probability, Likelihood Ratio, and Post-test Probability

**Figure 6:**
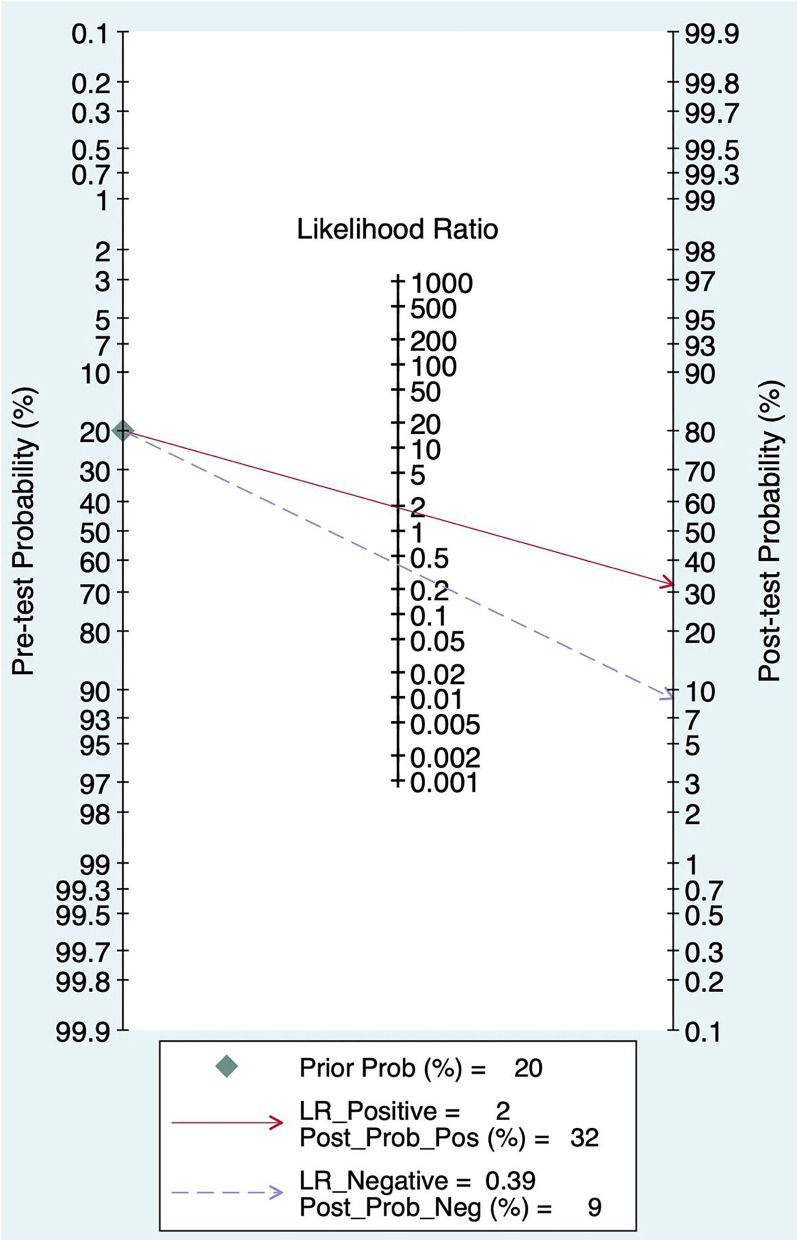
Fagan diagram of FAST in the diagnosis of AIS.

The Fagan graph was plotted to show the relationship among the prior probability, the likelihood ratio, and the posterior probability. The pretest probability was 20%, and the post-test probability of AIS was 32%. In addition, the PLR was <10 (PLR = 1.90), and the NLR was >0.1(NLR = 0.39), indicating that the diagnosis can neither be confirmed nor excluded. Their diagnostic value of FAST in AIS was limited, as shown in Figure 6.

### BEFAST Against AIS

**Figure 7:**
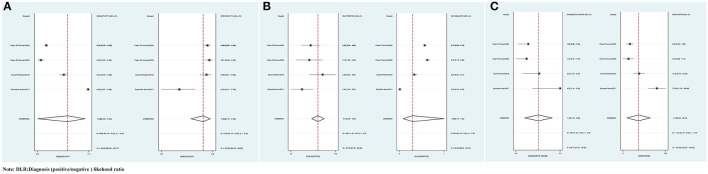
**(A)** Forest plot of Se and Sp of BEFAST in the diagnosis of AIS. **(B)** Forest plot of diagnosis (positive/negative) likehood ratio (DLR) positive and negative of AIS. **(C)** Forest map of the DOR of BEFAST in the diagnosis of AIS.

The combined Se was 0.68 [95% *CI* (0.23, 0.93)], Sp was 0.85 [95% *CI* (0.72, 0.92)], PLR was 4.41 [95% *CI* (3.48, 5.58)], NLR was 0.38 [95% *CI* (0.12, 1.25)], AUC was 0.86, and DOR was 11.49, which indicated that the BEFAST had a medium value in the screening of AIS. All heterogeneity was *I*^2^ > 50%; therefore, the random model was used in Figure 7.

### Publication Bias

**Figure 8:**
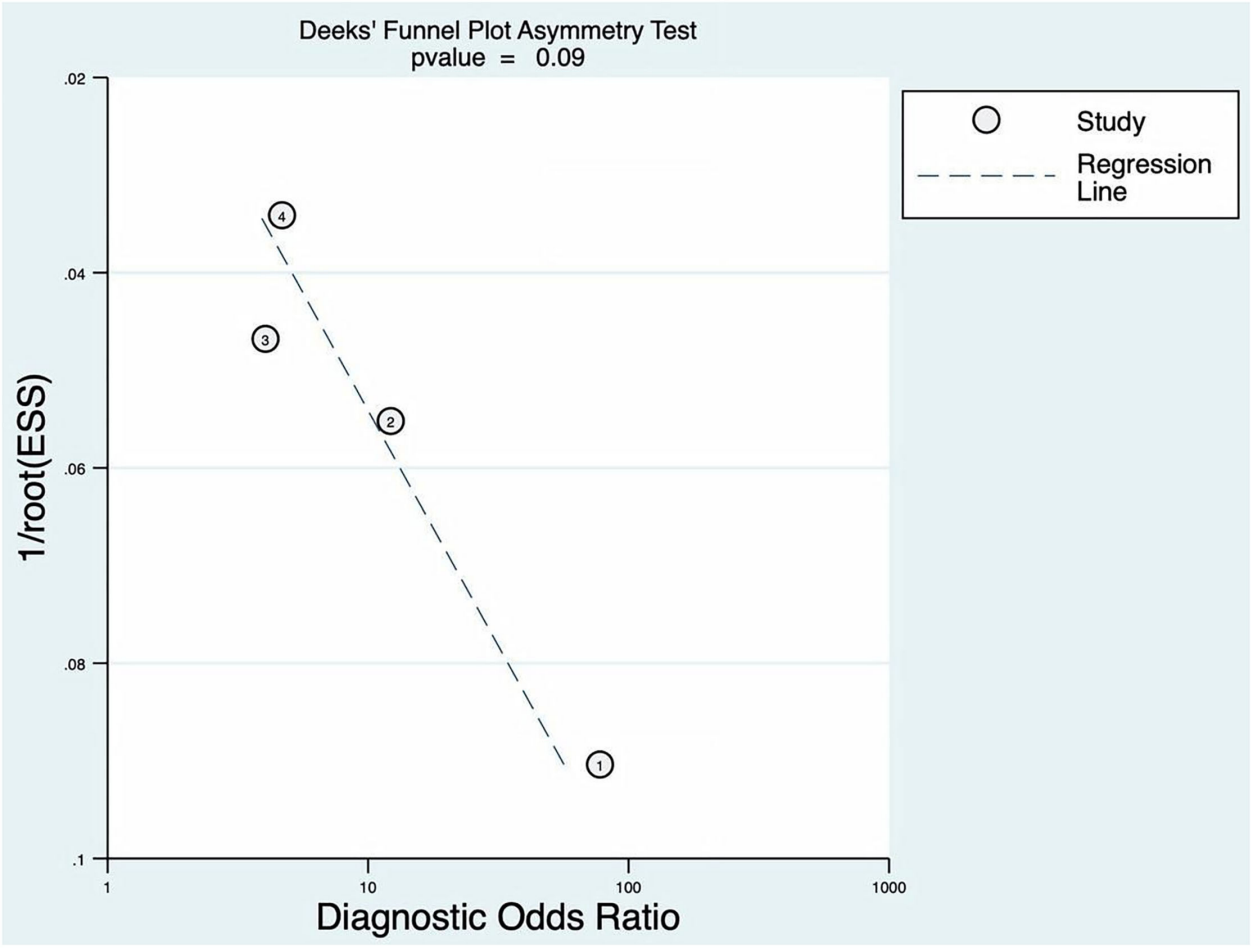
Deeks' funnel plot asymmetry test for BEFAST.

The *p* of Deeks' funnel plot asymmetry test was 0.09 (*p* > 0.05). There was no evidence of publication bias; the details are shown in Figure 8.

### Threshold Effect

**Figure 9:**
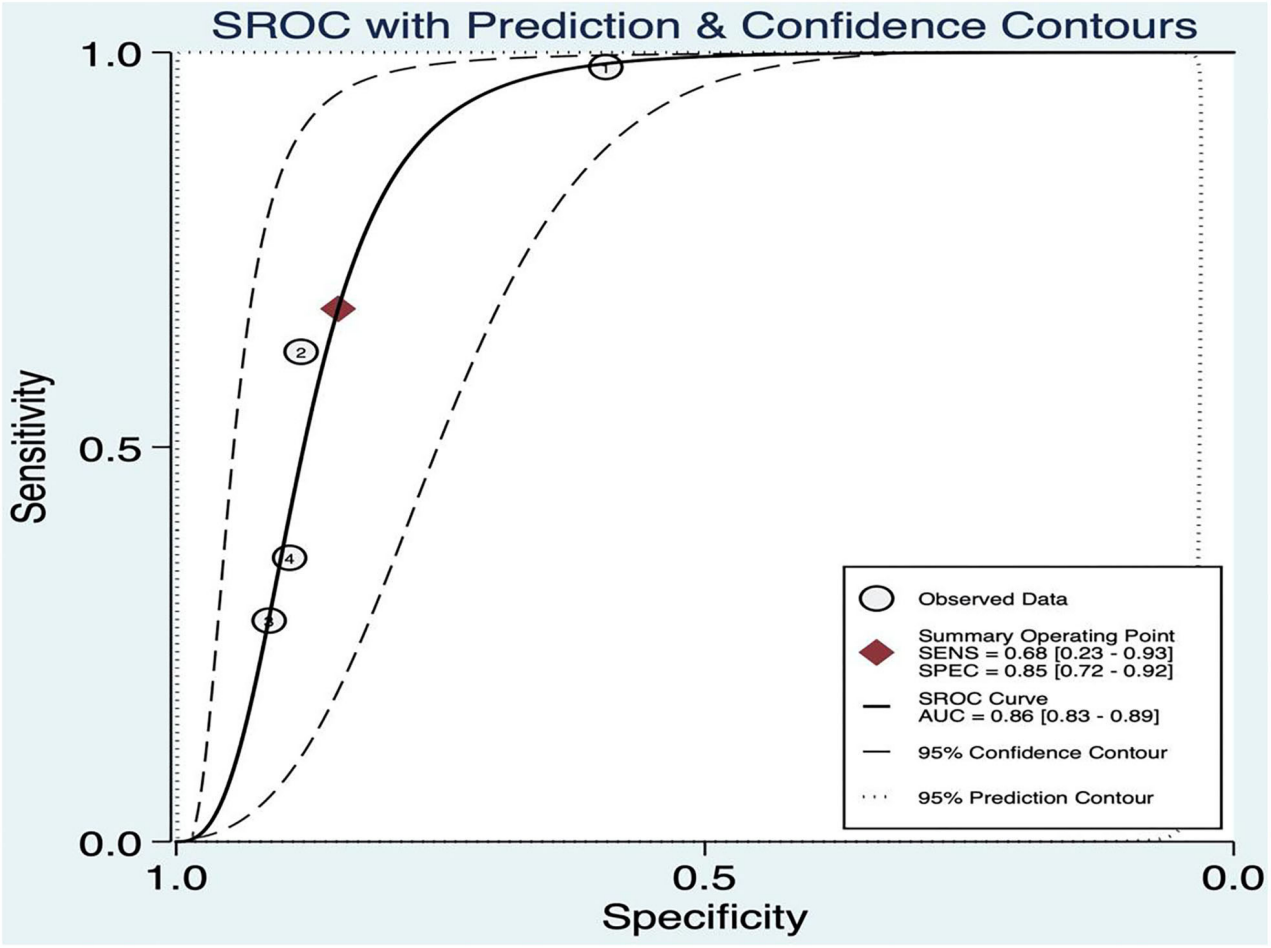
Summary ROC of BEFAST.

The threshold effect was assessed by the SROC curve plane test. As no typical “shoulder arm” was found, there was no threshold effect. A moderate diagnostic value was concluded by the value of the AUC, which was 0.86 [95% *CI* (0.83–0.89)]; details are shown in Figure 9.

### Pre-test Probability, Likelihood Ratio, and Post-test Probability

**Figure 10:**
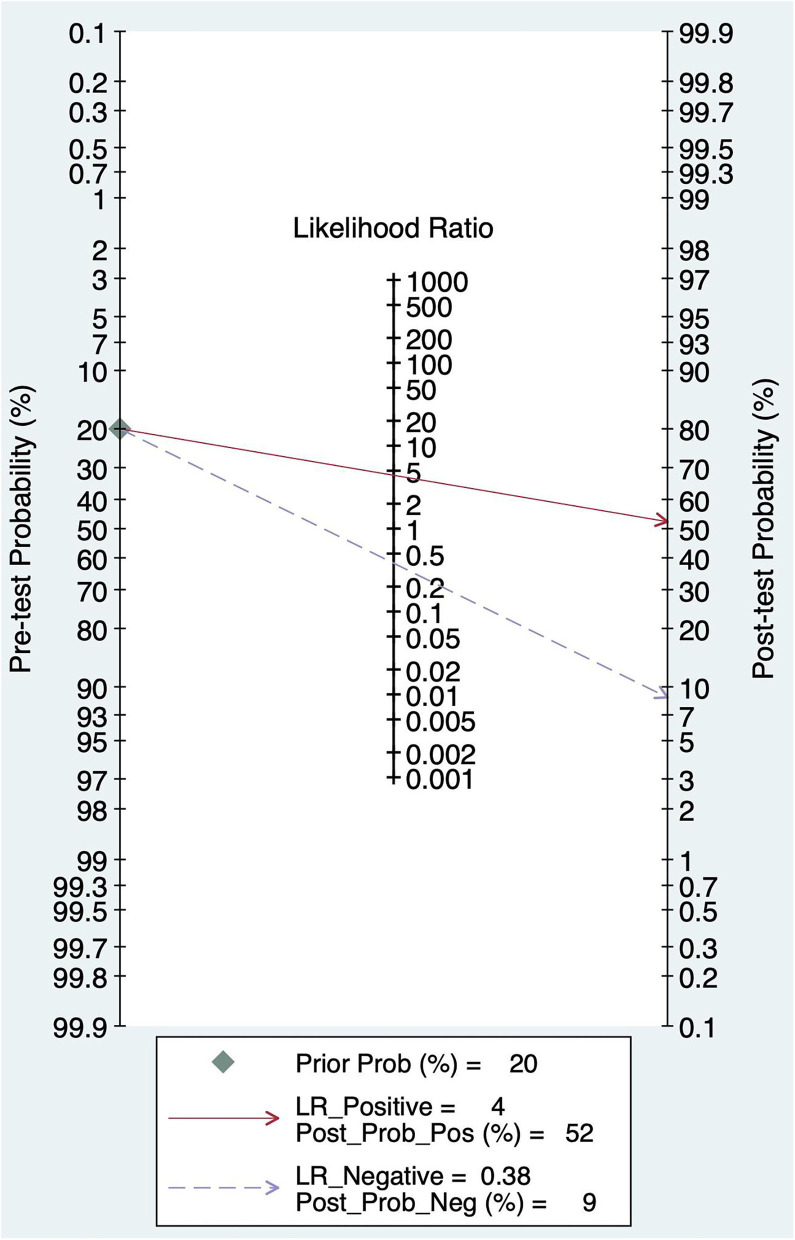
Fagan diagram of BEFAST in the diagnosis of AIS.

The pretest probability was 20%, and the probability of AIS was 52%. In addition, the PLR was <10 (PLR = 4.41), and the NLR was >0.1(NLR = 0.38), which indicated that the diagnosis could be neither confirmed nor excluded. Their diagnostic value of BEFAST in AIS was also limited; details are shown in Figure 10.

### Comparison of FAST, BEFAST, and FAST in Combination With BEFAST

Comparison of FAST, BEFAST, and FAST in combination with BEFAST was performed using ROC, Se, and Sp analysis. Among them, the BEFAST had the best diagnostic value; details are shown in Table 3.

**Table 3:** Diagnostic performance of FAST, BEFAST, and FAST in combination with BEFAST.

**Scale**	**Sensitivity**	**Specificity**	**AUC**	**Sensitivity combined**	**Specificity combined**	**Prior P**	**PLR (%)**	**NLR(%)**
FAST	0.77	0.60	0.76	0.74	0.69	20	32	9
BEFAST	0.68	0.85	0.86	0.68	0.85	20	52	9
FAST+BEFAST	0.74	0.69	0.78	0.74	0.69	20	37	9

*FAST, Face Arm Speech Test; BEFAST, Balance, Eyes, Face, Arm, Speech, Time; PLR, the positive likelihood ratio; NLR, the negative likelihood ratio*.

### Quality of All Studies

For prospective studies, the NOS scores varied from 6 to7 stars (Table 4A). For cross-sectional studies, the AHRQ scores varied from 4 to 6 (Table 4B).

**Table 4A:** Methodological quality assessments of included observational studies by Newcastle Ottawa scale (NOS).

**Study**	**Study design**	**NEWCASTLE - OTTAWA SCALE**		
		**Selection**	**Comparability**	**Exposure**
RT. Fothergill et al. (12)	Prospective cohort study	⋆⋆⋆⋆	⋆⋆	⋆✩✩
A. Berglund et al. (13)	Prospective cohort study	⋆⋆⋆⋆	⋆⋆	⋆✩✩
JC. Purrucker et al. (5)	Prospective cohort study	⋆⋆⋆⋆	⋆⋆	⋆✩✩
H. Mao et al. (11)	Prospective cohort study	⋆⋆⋆✩	⋆⋆	⋆✩✩
D. Pickham et al. (4)	Prospective cohort study	⋆⋆⋆⋆	⋆⋆	⋆✩✩
D. Václavík et al. (10)	Prospective cohort study	⋆⋆⋆⋆	⋆⋆	⋆✩✩

**Table 4B:** Methodological quality assessments of included cross-sectional studies by the Agency for Healthcare Research and Quality (AHRQ).

**Question**	**Define the source of information**	**List inclusion and exclusion criteria for exposed and unexposed subjects (cases and controls) or refer to previous publications**	**Indicate time period used for identifying patients**	**Indicate whether or not subjects were consecutive if not population-based**	**Indicate if evaluators of subjective components of study were masked to other aspects of the status of the participants**	**Describe any assessments undertaken for quality assurance purposes**	**Explain any patient exclusions from analysis**	**Describe how confounding was assessed and/or controlled**.	**If applicable, explain how missing data were handled in the analysis**	**Summarize patient response rates and completeness of data collection**	**Clarify what follow-up, if any, was expected and the percentage of patients for which incomplete data or follow-up was obtained**	**Score**
Answer	Yes (+) or no/unclear (–)	Yes (+) or no/unclear (–)	Yes (+) or no/unclear (–)	Yes (+) or no/unclear (–)	Yes (+) or no/unclear (–)	Yes (+) or no/unclear (–)	Yes (+) or no/unclear (–)	Yes (+) or no/unclear (–)	Yes (+) or no/unclear (–)	Yes (+) or no/unclear (–)	Yes (+) or no/unclear (–)	
WN Whiteley et al. (14)	**+**	**+**	**–**	**+**	**–**	**+**	**–**	**–**	**–**	**–**	**–**	4
S. Aroor et al. (7)	**+**	**+**	**+**	**+**	**+**	**–**	**–**	**–**	**–**	**–**	**–**	5
F. El Ammar et al. (8)	**+**	**+**	**+**	**+**	**+**	**–**	**+**	**–**	**–**	**–**	**–**	6

## Discussion

The phrase “time is brain” highlights that human nervous tissue is rapidly and permanently lost as stroke progress and that therapeutic intervention should be emergently pursued. Nonetheless, <10% of patients with stroke in hospitals undergo emergency treatment within the thrombolytic time window (15). Currently, stroke is a major cause of death and disability. The mean lifetime cost of ischemic stroke per person, which includes inpatient care, rehabilitation, and follow-up care, is expensive and unaffordable (16). Meanwhile, it affects the quality of life of patients and their families. Therefore, early recognition and accurate diagnosis are of essential importance for a positive outcome. In 1998, the FAST included a rapid ambulance protocol to improve the rapid triage of patients suspected of an acute stroke at our acute stroke unit (ASU) (17). Recently, it has been recorded that the ambulance services most commonly use the FAST to assess patients suspected of stroke (12).

Over recent years, the prehospital stroke scales have become increasingly used to assess acute stroke. Among them, FAST has the highest diagnostic value, with 88.9% of identified stroke/TIA patients within our population. However, the FAST failed to detect 38% of posterior cerebral circulation strokes (18, 19). Posterior circulation stroke, which represents 20~25% of patients with IS, is associated with a greater risk of disability and death compared with anterior circulation strokes (4). The FAST showed the ability to identify 69–90% of strokes, but it missed up to 40% of those with posterior circulation events. Missed diagnosis rates improved with the addition of visual symptoms and limb ataxia. Therefore, “B” was added for balance and an “E” for eyes (7). In 2020, Ammar et al. performed a retrospective analysis of inpatients screened with the stroke alert system and a final diagnosis of AIS, who were candidates for reperfusion therapy, revealing the Se of BEFAST to be 83% (20).

There has been an increasing number of Systematic reviews and meta-analyses assessing the diagnostic performance of clinical assessment over recent years. The previous systemic review and meta-analysis have evaluated the diagnostic value of the current common stroke identification scales worldwide. In 2014, a Systematic review showed that prehospital stroke scales varied in their accuracy, missing up to 30% of acute strokes in the field through the evaluation of FAST, CPSS, MASS, LAPSS Ontario Prehospital Stroke Screening Tool (OPSS), and Med PACS for diagnostic value with stroke in urban environment (21). In 2019, the assessment of both cortical and motor function using the Rapid Arterial Occlusion Evaluation Scale (RACE), Field Assessment Stroke Triage for Emergency Destination (FAST-ED) and National Institute of Health stroke scale (NIHSS) showed the best diagnostic accuracy values for selecting subjects with large vessel occlusion (LVO) (22). In 2020, a systematic review and meta-analysis revealed that ROSIER was a valid scale with high clinical applicability (23). Even though numerous scales have emerged for assessing the stroke, only a few studies compared the Se and Sp between FAST and BEFAST.

Our results showed that the FAST had higher Se than BEFAST in detecting AIS. By contrast, BEFAST had a higher Sp than FAST. In general, BEFAST had the highest diagnostic value; however, FAST, as well as BEFAST, may be useful in the diagnosis of AIS. Previous studies found that 14% of patients with AIS would be missed using FAST alone, and this proportion was reduced to 4.4% with the addition of a history of gait and visual symptoms (BEFAST). Our results were consistent with previous reports (7).

## Conclusion

Our findings indicated that FAST, as well as BEFAST, might be useful in the diagnosis of AIS; however, AIS could neither be confirmed nor excluded by the sole use of FAST or BEFAST. The diagnostic value of BEFAST in AIS was higher than FAST; thus, it might have an important role in the fast recognition of AIS. Nonetheless, it still remains unclear whether it could be applied for screening of all patients with stroke in the prehospital setting or in hospital, or whether the test characteristics of the FAST and BEFAST scales could be separately assessed for posterior and anterior circulation. Future prospective studies are needed to explore the diagnostic value of FAST and BEFAST in the anterior and posterior circulation, respectively, so as to improve the recognition rate of stroke, promote timely intervention, and reduce the burden on families and society.

### Study Limitation

First, there was moderate heterogeneity across studies, meta-regression, and subgroup analysis fail output due to the limited BEFAST data. Second, few included studies did not explicitly exclude participants. Both shortcomings should be further investigated and addressed by future studies.

## Data Availability Statement

The original contributions presented in the study are included in the article/supplementary material, further inquiries can be directed to the corresponding authors.

## Author Contributions

XC: perform the literature screening, data extraction, data analysis, results representation, and drafting the manuscript for intellectual content. XL: statistical analysis, interpreted the data, and contributed to and revised the manuscript for intellectual content. XW: study initiate and contribute to and revised the manuscript for intellectual content. LZ and YY: revised the manuscript. MG: electronic search and articles election. FX: instruct the detail steps for groups and draft the manuscript. XZ: perform the literature screening, data extraction, data analysis, and results representation. All authors contributed to the article and approved the submitted version.

## Funding

This work was supported by the National Natural Science Foundation of China, No. 8216050478 to XL, and 82073833 to XW; the Program from Yunnan Provincial Clinical Research Center for Neurological Diseases, No. 202002AA100204 to LZ and XL; the Basic Research Program of Yunnan Provincial Science and Technology Department, No. 202101AT070151, and 20190FE001(-222) to XL; Chengdu Science and Technology Bureau Focuses on Research and Development Support Plan, No. 2019-YF09-00097-SN to FX; the Popular Scientific Research Project of Sichuan Health Commission, No. 20PJ171; and the Yunnan education program, No. SYSX202036 to XL and FX.

## Conflict of Interest

The authors declare that the research was conducted in the absence of any commercial or financial relationships that could be construed as a potential conflict of interest.

## Publisher's Note

All claims expressed in this article are solely those of the authors and do not necessarily represent those of their affiliated organizations, or those of the publisher, the editors and the reviewers. Any product that may be evaluated in this article, or claim that may be made by its manufacturer, is not guaranteed or endorsed by the publisher.

## Footnotes

^1^The following variables used to adjust whether included studied met these criteria.
